# Assessment of cardio-oncology knowledge and practice among healthcare providers in Saudi Arabia: a comprehensive nationwide survey

**DOI:** 10.1186/s40959-024-00299-x

**Published:** 2024-12-31

**Authors:** Hisham A. Badreldin, Nada Alsuhebany, Lama Alfehaid, Mohammed Alzahrani, Maha Aldoughaim, Abdullah M. Alrajhi, Jumanah Alsufyani, Dania Elsherif, Kanan Alshammari

**Affiliations:** 1 Department of Pharmacy Practice, College of Pharmacy, King Saud bin Abdulaziz University for Health Sciences, Riyadh, Saudi Arabia; 2 Department of Pharmaceutical Care Services, King Abdulaziz Medical City, Ministry of National Guard Health Affairs, Riyadh, Saudi Arabia; 3 King Abdullah International Medical Research Center, Riyadh, Kingdom of Saudi Arabia; 4 Clinical Pharmacy Department, King Fahad Medical City, Riyadh, Saudi Arabia; 5 Department of Pharmacy Practice, AlFaisal University, Riyadh, Saudi Arabia; 6 College of Pharmacy, University of Medical Sciences and Technology, Khartoum, Sudan; 7 Department of Medical Oncology, Ministry of National Guard Health Affairs, Riyadh, Saudi Arabia

## Abstract

**Introduction:**

The evolving field of oncology necessitates effective management of cancer-related cardiovascular diseases. In Saudi Arabia, the incidence of cancer is rising, and there is a critical need for cardio-oncology services to address cancer treatment-related cardiovascular toxicity. This study aimed to evaluate the knowledge and practices of healthcare providers (HCPs) in Saudi Arabia regarding cardio-oncology.

**Methods:**

A cross-sectional study was conducted from January 2024 to April 2024 using an online survey targeting cardiologists, oncologists, and clinical pharmacists. The survey assessed demographics, perceptions of cardio-oncology, availability of services, and current practices. Data were analyzed using descriptive statistics, chi-squared tests, and bivariate analyses.

**Results:**

The survey received responses from 116 HCPs, including cardiologists (63.79%), oncologists (23.28%), and clinical pharmacists (12.93%). Most participants had over six years of experience, and only one had formal cardio-oncology training. While 84.48% recognized the importance of managing cardiac complications in cancer patients, only 42.24% were familiar with existing guidelines. Limited training programs and institutional resources were significant barriers to implementing cardio-oncology services. Despite agreement on the need for cardiotoxicity management, only one-third recommended cardioprotective agents as standard care.

**Conclusion:**

There is a notable deficiency in formal training and resources for cardio-oncology in Saudi Arabia. To bridge this gap, integrating cardio-oncology into training programs, establishing institutional guidelines, and adopting multidisciplinary care models are crucial. These measures will enhance the quality of care for cancer patients and improve their cardiovascular outcomes.

## Introduction

The oncology field is witnessing a rapid evolution in systemic therapy regimens and guidelines globally [1]. In 2020 alone, 17,631 newly diagnosed cases of cancer were reported to the Saudi Cancer Registry, with the most common being breast, colorectal then thyroid cancers [2]. With improvements in cancer management leading to reduced cancer-related mortality rates, the management of cardiovascular diseases and accessibility to cardiovascular care have become increasingly paramount [3]. Despite advancements in medical care, cancer and cardiovascular diseases remain prominent causes of mortality globally [4].

Cancer and cardiovascular diseases share similar risk factors, such as increasing age, smoking, alcohol use, and high BMI [5–8], this contributes to the rising prevalence of both diseases. This is further complicated by the fact that it has been well-established that cancer treatments can cause cancer treatment-related cardiovascular toxicity (CTR-CVT) [9, 10]. Since then, it has been suggested that cardiotoxic cancer medications can be divided into drugs that can potentially cause irreversible damage (Type I) and drugs that predominantly induce reversible dysfunction (Type II) [11].

Cardio-oncology has emerged as an expanding specialty dedicated to providing cardiovascular care for both active cancer patients and cancer survivors in direct response to the increasing requirements of this population [1]. The scope of cardio-oncology encompasses optimizing pre-cancer treatment, diagnosing, and managing cardiac complications resulting from cancer treatment both during and after the completion of cancer therapy. Although numerous cardio-oncology programs have emerged to address these demands, they remain predominantly confined to larger institutions, often serving as tertiary care academic referral centers.

Over the years, several international organizations, including the American Society of Clinical Oncology (ASCO), the European Society of Cardiology (ESC), and the National Comprehensive Cancer Network (NCCN), have issued several position statements and guidelines pertaining to the detection and management of cancer treatment-related cardiac dysfunction (CTRCD) [11–13]. However, cardio-oncology is still an emerging field. Due to a lack of training programs, it is unclear how well-trained healthcare providers (HCPs) are in this subspecialty. In a survey of 106 cardiovascular specialists in the United States, published in the Journal of American College of Cardiology, over 43% of respondents had no dedicated cardio-oncology training programs for cardiology fellows in their institutions [14].

Notably, in Saudi Arabia, cardio-oncology programs have been limitedly implemented thus far, and few to no formal training programs in the field are available. Thus, this research aims to assess the practical knowledge of HCPs, including cardiologists, oncologists, and clinical pharmacists, on cardio-oncology in Saudi Arabia. This would inform healthcare providers on how to better improve the quality of life of their patients and the care provided to them.

## Methods

### Study design and subjects

A cross-sectional study was conducted in Saudi Arabia between January 2024 and April 2024. An online survey was created in English using Google Forms and distributed via email and social media platforms such as WhatsApp. The survey targeted physicians and clinical pharmacists specializing in oncology or cardiology. The survey was accompanied by a cover letter that described the research study’s objective and targeted population, and participants were given access to the survey after accepting participation. The survey was created by two oncologists and two cardiologists specializing in cardio-oncology. It was previously validated and used internationally in a study conducted by Peng et al. in 2019. The survey was obtained with the corresponding author’s permission [15]. The study received Institutional Review Board (IRB) approval from King Abdullah International Medical Research Center at National Guard Health Affairs in Riyadh, Saudi Arabia (IRB/2930/23).

### Questionnaire items

The survey comprised seven sections, including multiple-choice and Likert scale questions, as well as two clinical scenarios. It contained a total of 45 questions, 13 of which were for oncologists and five for cardiologists. The first section collected the participants’ demographic information. The second section evaluated the participants’ perceptions of cardio-oncology, for instance, inquiring about one’s definition of “cardio-oncology”. The third section assessed the availability of cardio-oncology services at participants’ institutions. The fourth section gathered opinions regarding the current practices in cardio-oncology. It aimed to evaluate the perceived importance of oncologists in considering cardiotoxicity during different stages of cancer therapy, such as planning, utilization, and completion, and how it affects their decision-making processes. It also assessed participants’ opinions on whether cancer patients would benefit from cardiologists’ involvement and cardio-oncology clinics. The fifth section of the questionnaire is directed explicitly to cardiologists. This section assessed cardiologists’ knowledge and comfort level in recognizing and managing cardiotoxicity. Furthermore, it aimed to determine the cardiologists’ perception of the skills and expertise possessed by their oncology colleagues in managing cardiotoxicity-related issues. The following section of the questionnaire was designed solely for oncologists to assess their knowledge and comfort level in identifying and managing cardiotoxicity. In addition, it aimed to determine their perspective on the skill of their cardiology colleagues in dealing with cardiotoxicity-related cancer therapy. The last section presented two clinical scenarios of common cardiotoxicity issues in oncology to assess participants’ ability to apply their knowledge.

### Statistical analysis

Descriptive quantitative analysis was generated via the “response” feature in Google Forms. Chi-squared test was used to compare categorical variables, and bivariate analysis was used to examine associations between the dependent and independent variables. The response rate was not obtainable, given the survey was distributed online. Statistical analyses were conducted using Excel software version 16.88.

## Results

### Demographics of respondents and experience in practice

A total of 116 HCPs in Saudi Arabia participated in the survey. Most respondents were cardiologists (63.79%), followed by oncologists (23.28%), and clinical pharmacists specializing in cardiology and oncology (12.93%). Nearly more than half of the respondents were from the central region of Saudi Arabia (67.24%). The majority of participants had over six years of experience (80.68%). Out of the respondents, only one physician received formal training in cardio-oncology. Additionally, 87.07% of the practitioners worked in tertiary hospitals (Table 1).

**Table 1:** Demographics of healthcare professionals surveyed (*n* = 116)

Region	*n*	%
Central region	78	67.24
Eastern region	18	15.52
Western region	11	9.48
Northern region	4	3.45
Southern region	5	4.31
**Specialty**		
Cardiology physician	74	63.79
Oncology/hematology physician	27	23.28
Clinical pharmacist in cardiology field	8	6.90
Clinical pharmacist in oncology/hematology field	7	6.03
**Current position**		
Consultant (physician or pharmacist)	88	48.35
Staff physician	8	4.40
Senior pharmacist	5	2.75
Fellow	12	6.59
Medical Resident	3	1.65
**Years practice of consultant (physician or pharmacist)**		
0–5 years	17	19.32
6–10 years	19	21.59
11–20 years	32	36.36
> 20 years	20	22.73
**Practice location description**		
Tertiary care hospital	101	87.07
Secondary hospital	11	9.48
Private clinic/hospital	4	3.45
**Percentage of time spent on clinical research**		
Less than 10%	60	51.72
10–20%	31	26.72
20–30%	15	12.93
30–40%	4	3.45
40–50%	1	0.86
More than 50%	5	4.31

### Perception of cardio-oncology and cardiotoxicity

**Fig. 1:**
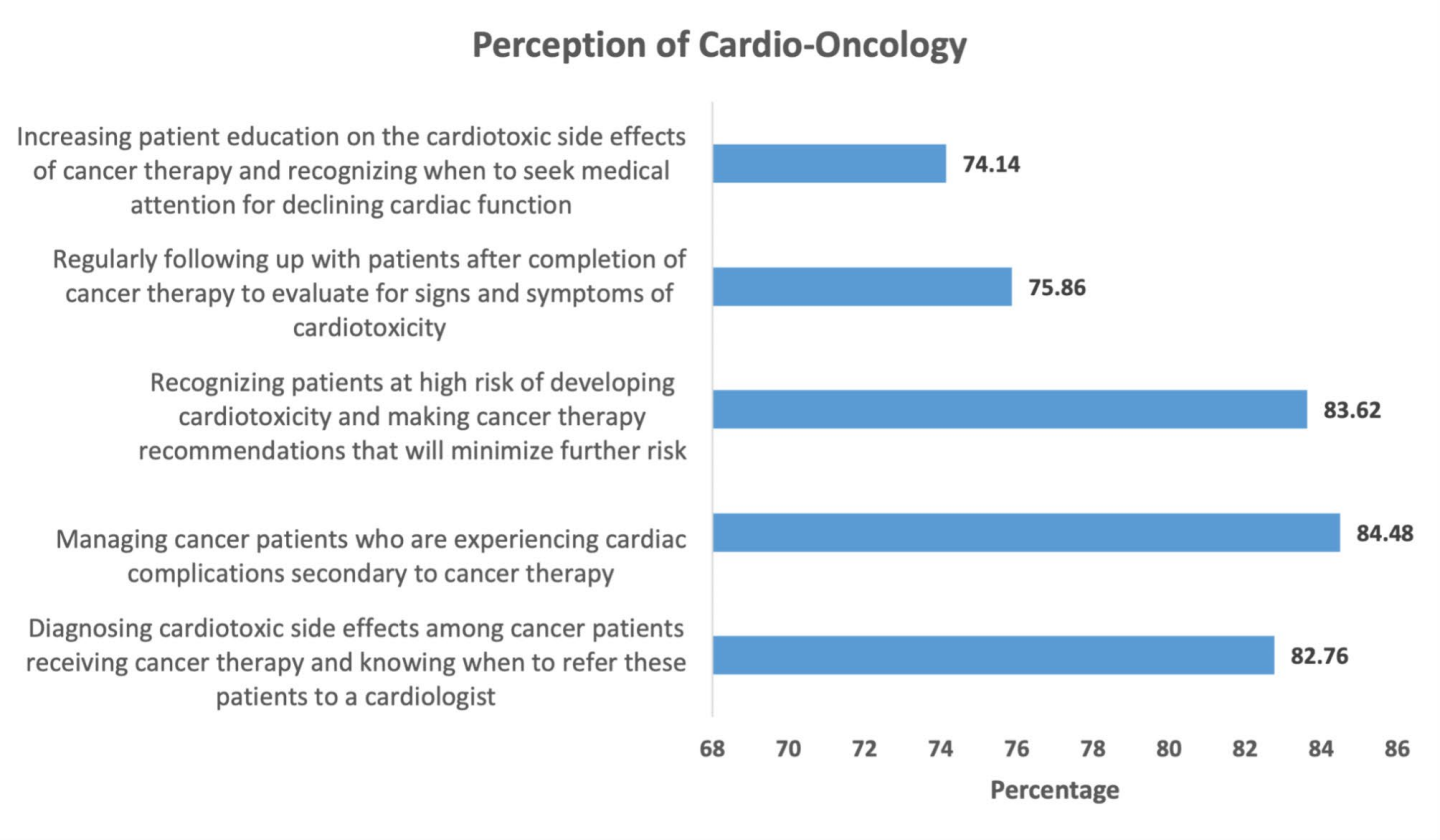
The percentage of perception of cardio-oncology by respondents

Regarding the HCP’s perception towards cardio-oncology, the respondents had the chance to respond to this question with multiple choice questions where multiple answers were accepted. The majority of respondents believed cardio-oncology means managing cancer patients who are experiencing cardiac complications secondary to cancer therapy (84.48%). Furthermore, more than (80%) believed cardio-oncology is about recognizing patients at high-risk of developing cardiotoxicity from cancer treatment and diagnosing cardiotoxicity induced by cancer treatment (83.62 and 82.76%, respectively) (Fig. 1).

Regarding the HCPs perception towards the most significant risk of cardiotoxicity induced by cancer treatment, 70.69% of respondents believe that targeted agents (such as trastuzumab) induced cardiotoxicity could be experienced during cancer treatment. In addition, 56.90% believe that chemotherapy-induced cardiotoxicity could develop during cancer treatment (Table 2).

**Table 2:** Perceptions of cardiotoxicity risk

**The greatest risk of experiencing cardiotoxicity from chemotherapy (e.g. anthracyclines),** ***n*** **(%)**	
During cancer treatment (chemotherapy and/or targeted agents)	66 (56.9)
One-year post-cancer therapy (short-term risk)	42 (36.21)
One to five years post-cancer therapy	48 (41.38)
Greater than five years post-cancer therapy (long-term risk)	52 (44.83)
**The greatest risk of experiencing cardiotoxicity from targeted therapies (e.g. trastuzumab)**,** n (%)**	
During cancer treatment (chemotherapy and/or targeted agents)	82 (70.69)
One-year post-cancer therapy (short-term risk)	26 (22.42)
One to five years post-cancer therapy	39 (33.63)
Greater than five years post-cancer therapy (long-term risk)	32 (27.59)

**Fig. 2:**
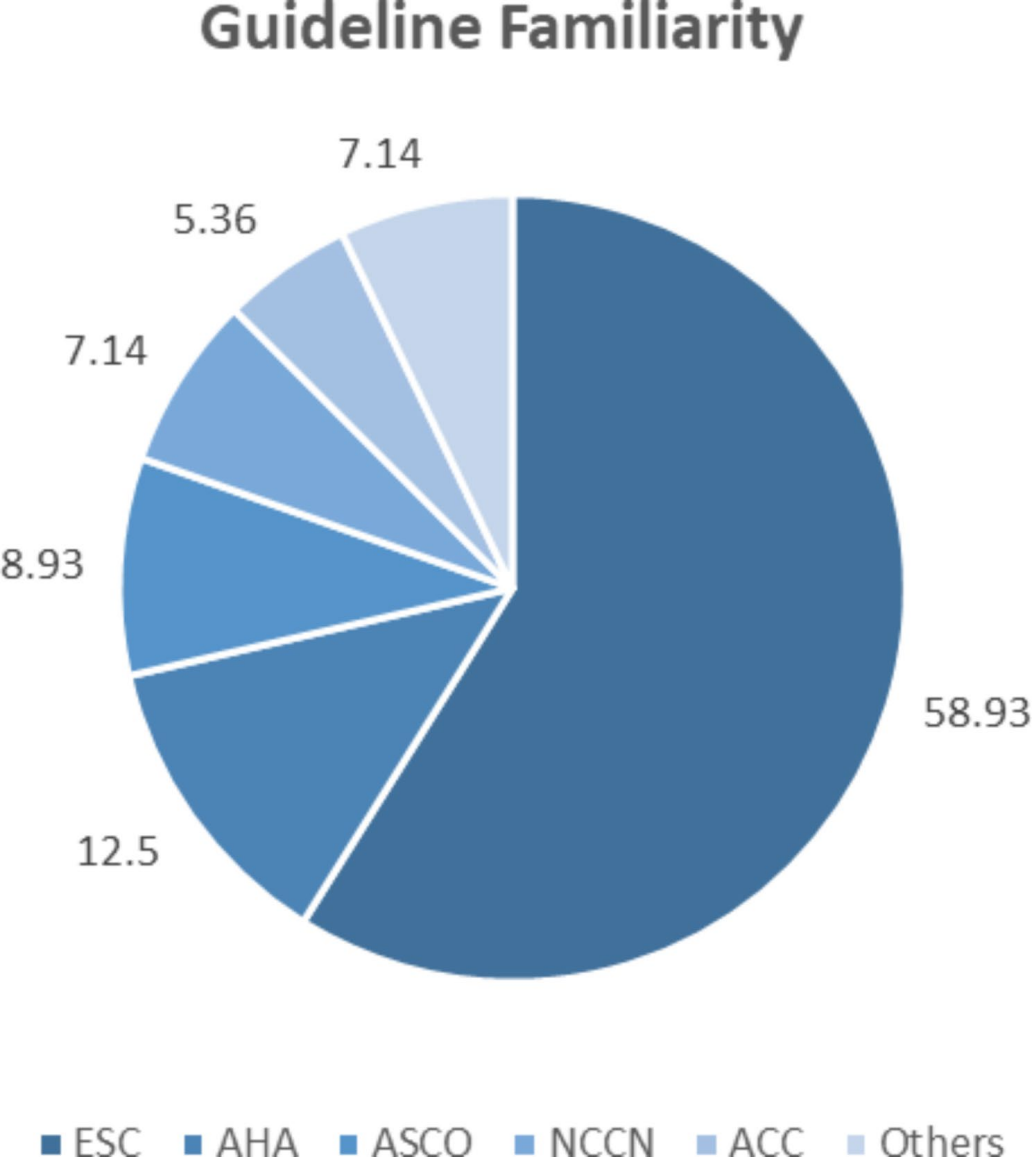
The percentage of guidelines familiarity reported by respondents

Almost 42.24% of respondents reported being familiar with guidelines from expert societies on the directions and management of cardiovascular toxicity. Out of the reported societies, the ESC was the most recognized guideline (58.93%) by the respondents (Fig. 2).

### Availability of cardio-oncology services at respondents’ institution

This survey assessed the availability of educational programs in cardio-oncology. The most frequent answer regarding educational programs was “there is little cardiology training for clinical cardiology fellows” (36.21%). Interestingly, 31.9% of respondents reported the lack of program availability at all. Regarding cardio-oncology services offered currently at respondents’ institutions, almost half of the respondents reported providing such services on a consultation-based model (55.18%). Reflecting on the potential obstacles to the development of cardio-oncology units, “limited interest” was the most frequent answer (40.52%) reported by the respondents, followed by “limited funding” and “limited infrastructure” (28.45%, 25%) (Table 3).

**Table 3:** Availability of cardio-oncology services at respondents’ institution

**Education programs are available in the field of cardio-oncology,** ***n*** **(%)**	
There is little cardio-oncology training for clinical cardiology fellows	42 (36.21)
Cardiology fellows are exposed to cardio-oncology during clinical rotations as part of the curriculum	9 (7.76)
There are some lectures on cardio-oncology as part of the core curriculum or CME offerings	22 (18.97)
Cardiology fellows can choose to spend time training with the oncology service, but this is not part of the core curriculum	6 (5.18)
There is a dedicated cardio-oncology fellowship program (at least 6 months)	1 (0.87)
I am unsure of available cardio-oncology programs	38 (32.76)
No available programs at all	37 (31.9)
**Cardio-oncology services are currently offered**	
Consultation service operated by general cardiologists for assessment and management of cardiotoxicity among cancer patients	64 (55.18)
Physicians specialized in the field of cardio-oncology who are comfortable managing cardio-toxic side effects due to cancer therapy and can give recommendations for adjusting cancer therapy	3 (2.59)
Clinical pharmacists in the field of cardio-oncology who are comfortable managing cardio-toxic side effects due to cancer therapy and can give recommendations for adjusting cancer therapy	5 (4.32)
Cardio-oncology services are not currently present and there are no plans to add these services	10 (8.63)
Cardio-oncology services are not currently present but there are plans to add these services within the year	7 (6.04)
I am unsure of the available services	27 (23.28)
**Potential obstacles to the development of cardio-oncology units**	
Limited funding	33 (28.45)
Limited infrastructure	29 (25)
Limited need	7 (6.04)
Limited interest	47 (40.52)
No obstacle	32 (27.59)

### Respondents’ opinions towards current practice

**Fig. 3:**
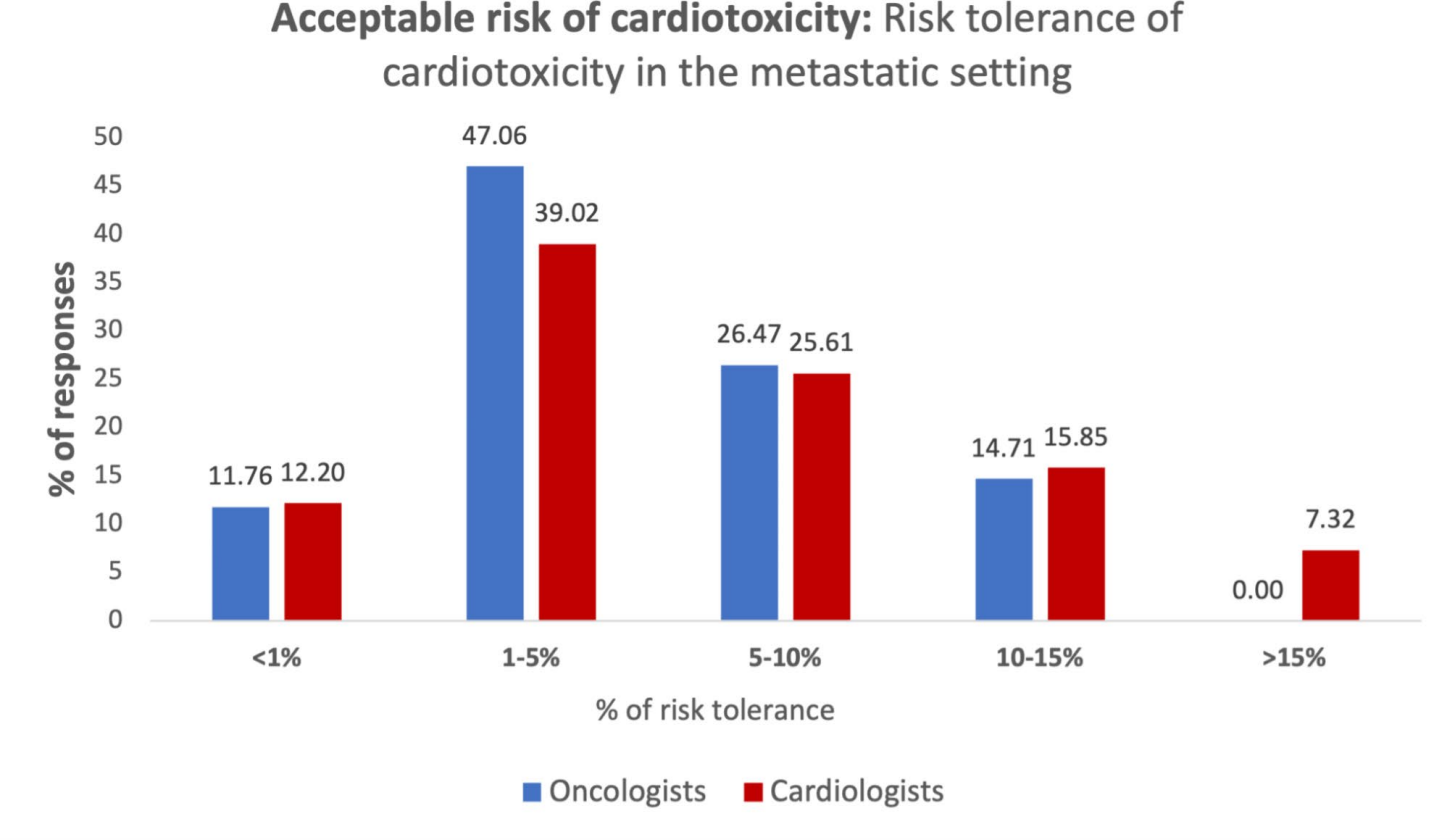
Acceptable risk of cardiotoxicity: risk tolerance of cardiotoxicity in the metastatic setting

In the current study, both cardiologists and oncologists agreed on the importance of oncologists’ consideration of cardiotoxicity when planning treatment plans (82.93% and 79.41%, respectively). Furthermore, both specialist groups agreed on the importance of monitoring for possible cardiac adverse events during active cancer treatments (85.37% and 76.47%, respectively). For cancer survivors, both groups agreed that oncologists should consider possible cardiac complications induced by cancer treatment exposure (58.54% and 55.88, respectively). Half of the respondents believed in the involvement of cardiologists in providing ongoing monitoring for cardiotoxicity, even if the patient has no clinical symptoms of cardiac issues. On a scale of 5, 34.15% of cardiologists strongly agree on using cardioprotective agents as a standard of care while 35% of oncologists were neutral about this statement. The majority of respondents (87.93%) thought that access to cardio-oncology services would not change the prognosis of cancer patients. In the setting of metastatic cancer, more cardiologists (7.32%) accepted a higher risk of cardiotoxicity (> 15% risk) compared to oncologists (0%) (Fig. 3), However, both oncologists and cardiologists accepted cardiotoxicity ranging between 5 and 15%.

### Current practice of respondents

Cardiologists’ responses to the statement “I am knowledgeable about cardiovascular complications of cancer therapy” were mostly neutral (41.46%) followed by agreement (37.80%). There was an agreement among cardiologists about their comfort in treating cardiovascular complications induced by cancer therapy, where most of them responded as neutral, agree, and strongly agree (28.05%, 32.93%, and 25.61%, respectively). In addition to that, cardiologists’ responses were mostly neutral when asked how oncologists are knowledgeable and comfortable with managing cardiovascular toxicities of cancer therapy (42.68% and 31.71%, respectively). Almost 40% of cardiologists reported that they sometimes prescribe cardiac medications to manage patients with cardiotoxicities as a result of cancer therapy.

When it comes to oncologists’ practice, almost 70% of oncologists feel comfortable treating cardiovascular toxicities induced by cancer therapy. In addition to that, 50% of them feel comfortable managing such cases. Furthermore, 44.12% of oncologists were neutral when asked “whether cardiologists are knowledgeable about these toxicities” and 35.29% agreed that cardiologists are comfortable managing such cases. Moreover, 33.8% of oncologists responded that they ask all cancer patients about cardiovascular risk factors prior to starting any cancer therapy. Similar to cardiologists, almost 40% of oncologists reported that they sometimes prescribe cardiac medications to patients with cardiovascular toxicities.

## Discussion

The advancements in cancer therapy in recent years have contributed to increased overall survival in various cancer types, resulting in a population with emerging medical needs, such as cardio-oncology care [16]. A few studies have been conducted to describe the cardio-oncology landscape among patients with cancer in Saudi Arabia [17, 18]. A single-center retrospective analysis that was conducted in Najran identified cardiac toxicity in around 15% of their cancer patient population [17]. Similarly, a larger retrospective analysis by Aljazairi and colleagues included over 1000 patients who received trastuzumab, pertuzumab, and bevacizumab; cardiovascular toxicities were detected in around 16% of their patient population [18]. A recent guideline update by the Saudi Heart Association expanded recommendations to focus on special populations, including oncology patients, to further accommodate the growing needs of patients living with cancer [19]. To our knowledge, this study is the first to capture the current state of cardio-oncology practices among healthcare providers in Saudi Arabia to help address knowledge gaps, educational and training needs in the country.

Our survey was completed by 116 HCPs, most of whom were cardiology specialists. Most respondents had over five years of clinical experience and practiced in tertiary care institutions. Notably, out of the 116 responders, only one physician received formal cardio-oncology training, which reflects the current limited state of exposure to the field during residency and fellowship training. Most respondents were able to identify the growing role of cardio-oncology care and the major cardiovascular offenders, such as trastuzumab and anthracyclines. On the other hand, less than half of the respondents identified relevant information sources to refer back to, which is a considerable limitation to the provision of cardio-oncology care. This can be improved by establishing institutional practice guidelines and introducing national and international practice guidelines in the field of cardio-oncology in training programs and academic curriculums. The American College of Cardiology established a framework for cardio-oncology subspecialty fellowship programs; it outlines the different levels of competency a program should offer, which range from a basic understanding of cardio-oncology concepts to rigorous coverage in cardio-oncology specialized clinics [20]. Multiple healthcare systems in the United States have shared successful experiences in implementing specialized training programs; several aspects can be adopted in Saudi Arabia, such as cross-training in specialized institutions and increasing focus on multidisciplinary care models [21, 22].

The survey of our study was adopted from Peng et al.’s study, hence we compared our findings to Peng et al. results [15] which revealed several important similarities, and differences. While our study focused on a specific region in the Middle East, Saudi Arabia, and targeted cardiologists and oncologists, Peng et al.‘s study had a broader geographic scope, including participants from 22 countries across multiple continents. This broader scope allowed Peng et al. to capture a more diverse range of practices and opinions, while our study provided a more focused insight into the cardio-oncology landscape within Saudi Arabia. In terms of demographics, both studies reported a higher participation rate among cardiologists than oncologists. When comparing perceptions of cardio-oncology and cardiotoxicity, both studies revealed that the majority of respondents recognized the importance of managing cardiac complications secondary to cancer therapy and the need for cardiologists’ involvement in cancer care. However, Peng et al. found a more significant disparity between cardiologists and oncologists regarding when and how cardiologists should be involved in cancer patient care. Moreover, the availability of cardio-oncology services and training programs was a common concern in both studies. Peng et al. highlighted limited funding and infrastructure as significant barriers to the development of cardio-oncology clinics globally, whereas our study found that limited interest was a major obstacle in Saudi Arabia followed by financial obstacles.

Furthermore, the majority of respondents of our study have highlighted the limited cardio-oncology services at their respective institutions. The barriers to establishing a specialized service were thought to be limited interest and financial obstacles. Recommendations from a consolidated framework for implementation research (CFIR) model performed at an academic medical center in the United States may be utilized to help guide the implementation of cardio-oncology services in Saudi Arabia; this includes standardization of referral processes and integrating decision-making tools in electronic health records [23]. Another positive experience in implementing specialized cardio-oncology services was reported by the Iraqi Cardio-Oncology Program-Pharmacist (ICOP-Pharm). The ICOP model integrates experienced clinical pharmacists in direct patient care as a part of a multidisciplinary care team; this allowed better drug therapy optimization and helped cardiologists manage a larger number of patients [24].

Metastatic cancer disease survival has increased significantly over the last few years in which common cancer diseases such as metastatic colorectal, and breast cancers have median survival of more than 2–3 years [25, 26]. The results of our study indicate that in the setting of metastatic cancer, a higher percentage of cardiologists were willing to accept a cardiotoxicity risk greater than 15%, whereas none of the oncologists were willing to accept this level of risk. This finding is particularly noteworthy as it suggests that oncologists may be less willing to accept higher cardiotoxicity risks in these patients because many of them can maintain a good quality of life during their extended survival period. In contrast, cardiologists might be more inclined to accept higher risks, potentially due to limited awareness of the significant improvements in survival rates and quality of life of metastatic cancer patients. This difference in perception demonstrates the need for better interdisciplinary communication and education to align treatment goals and risk assessments between cardiologists and oncologists, ensuring that patients receive the most appropriate care tailored to their long-term survival prospects.

Survey domains evaluating current practices have shown a general agreement on considering potential cardiotoxicities in oncology patients and the involvement of cardiology in managing patients who develop toxicities. However, one-third of respondents actively recommend cardioprotective agents in the care process. The described practice is consistent with the ESC cardio-oncology guidelines, which suggests only considering cardioprotective strategies in high-risk subsets of patients based on small clinical trials [12]. Surprisingly, over 80% of responders thought that cardio-oncology care will not change cancer prognosis. In addition, there seems to be a gap in cardiovascular risk factor screenings in oncology patients undergoing therapy. These interesting results indicate that cancer and cardiovascular disease are viewed as separate disease states rather than a part of the oncology disease continuum; this further confirms the need for robust cardio-oncology services since several large studies have shown increased cardiovascular disease incidence and mortality in patients with cancer compared to the general population [16, 27–34].

This nationwide study has several limitations. First, the survey was distributed on a large scale via link distribution, limiting our ability to assess the survey response rate. Moreover, the majority of respondents were practicing in academic and tertiary care centers in the central region of Saudi Arabia, which limits the generalizability of these results to smaller healthcare settings. We also could not assess regional differences across Saudi Arabia since most respondents were from the central region, which is another limitation to the generalizability of the results. In addition, there is a component of participation bias as the opinions of this survey were primarily reflected from the cardiologists rather than the oncologists even though equal distribution to both fields were ensured. However, this might be due to that cardiologists are more enthusiastic about cardio-oncology field since this field is usually led by cardiologists. Last, this survey does not capture the perspective of primary care practitioners who usually have frequent contact with patients requiring cardio-oncology care.

## Conclusion

The study highlights a significant knowledge gap and limited exposure to cardio-oncology among HCPs in Saudi Arabia. Despite recognizing the importance of managing cardiotoxicity in cancer patients, formal training and comprehensive cardio-oncology services remain limited. Implementing institutional guidelines, integrating cardio-oncology into medical education, and adopting multidisciplinary care models are essential to improve patient outcomes and address the growing needs of cancer patients at risk of cardiovascular complications.

## Data Availability

The data that support the findings of this study are available from the corresponding author on reasonable request.
